# AI-Related Stress and Suicidal Tendencies in a Primary Care Cohort: Clinical Audit

**DOI:** 10.2196/84661

**Published:** 2026-07-29

**Authors:** Shemarah Lindo

**Affiliations:** 1 Independent Researcher, Trinity, Port Maria, St. Mary, Jamaica, +1 876 434 7182

**Keywords:** artificial intelligence, mental health, technostress, AI anxiety, clinical audit, chatbots, suicidal ideation, Nigeria

## Abstract

Rapid AI integration has introduced novel psychosocial stressors. Little is known about AI-specific clinical impacts in resource-limited settings. This study aimed to assess AI-associated distress prevalence and nature in a Nigerian primary care and psychiatry clinic. Retrospective audit of 28 consecutive patients with anxiety, depression, or stress (April–August 2025) at J-Shalom Hospital, a primary care and psychiatry clinic in Ibadan. Technology-related stressor questions adapted from the AIAS (Artificial Intelligence Anxiety Scale) were incorporated into routine clinical interviews; C-SSRS (Columbia-Suicide Severity Rating Scale) evaluated suicidality as part of standard clinical practice. A total of 67.9% (19/28; 95% CI 49.3%‐82.1%) reported technology stress; 42.9% (12/28; 95% CI 26.5%‐60.9%) identified AI-specific stressors. Chatbot distress was most common (n=7/12, 58.3%). Three patients (25.0%; 95% CI 8.9%‐53.2%) reported worsening suicidal ideation following distressing chatbot interactions characterized by perceived rejection or invalidation. AI stressors are emerging clinical presentations. The chatbot–suicidality link demands urgent regulatory attention.

## Introduction

Health care digitization has expanded accessibility, yet generative AI’s rapid proliferation has introduced complex psychosocial stressors. While social media’s impact on anxiety is established [1], AI interactions’ psychological effects termed ’AI anxiety’ or technostress remain understudied [2,3]. The Artificial Intelligence Anxiety Scale (AIAS) conceptualizes AI-related anxiety as multidimensional [4], with recent research revealing that algorithmic opacity and adaptation demands can exacerbate anxiety and depression [5,6].

Conversational agents present particular concerns. Despite being marketed as mental health supports, chatbots are prone to hallucinations and sycophancy, with growing reports linking system failures to suicidal ideation [7,8]. Given limited Global South clinical data, this audit assessed AI-related stressor prevalence in a Nigerian clinic.

## Methods

### Study Design

Retrospective audit of consecutive patients presenting with anxiety, depression, or stress (April–August 2025) at J-Shalom Hospital, a primary care and psychiatry clinic in Ibadan, Nigeria. The clinic operates part time psychiatric services and therefore records a relatively low volume of mental health consultations during the audit period. Of 38 mental health presentations, 28 completed screening.

### Ethical Considerations

Per Nigeria’s National Code of Health Research Ethics (NCHRE) [9], clinical audits evaluating existing care practices without experimental intervention are exempt from full Health Research Ethics Committee review. Data were collected as part of routine clinical assessment and subsequently anonymised for audit purposes. Screening questions related to technology stress were incorporated into routine clinical interviews conducted by treating clinicians. No identifiable patient information was retained. Permission to review anonymised clinic records for audit purposes was granted by J-Shalom Hospital administration. The clinic operates under the purview of the Federal Ministry of Health guidelines; no formal data transfer agreement or external institutional approval was required for internal audit of routinely collected data.

### Participants

- Inclusion criteria: age ≥18 years, chief complaint of anxiety, depression, or stress, ability to communicate in English or Yoruba
- Exclusion criteria: acute psychosis, severe cognitive impairment, refusal of consent to routine data use

Consent refusal was treated as an exclusion criterion consistent with standard clinical practice; no experimental procedures requiring formal research consent were conducted. Of 38 eligible patients, 7 declined routine clinical data use or were excluded on clinical grounds, yielding a final sample of 28.

### Data Collection

Two clinicians conducted screening using AIAS-adapted questions incorporated into routine clinical interviews, covering general technology stress and four AI dimensions (learning/interaction, job replacement, sociotechnical, and ethical/configuration anxiety). Suicidality was assessed via the Columbia–Suicide Severity Rating Scale (C-SSRS) as part of standard intake. These instruments were used as clinical tools, not research instruments administered outside usual care.

### Analysis

95% CIs were calculated using Wilson’s score method appropriate for small samples (n<30) [10].

## Results

Of 28 patients (mean age 32.4, SD 12.1; 60.7% female), 67.9% (19/28; 95% CI 49.3%‐82.1%) reported technology stress; 42.9% (12/28; 95% CI 26.5%‐60.9%) identified AI-specific stressors. Some patients reported more than one AI-related stressor category. Among AI-stressed patients, chatbot distress dominated (n=7, 58.3%), followed by job displacement anxiety (n=3, 25.0%) and health misinformation distress (n=2, 16.7%). Three patients (n=3, 25.0%; 95% CI 8.9%‐53.2%) reported worsening suicidal ideation following distressing chatbot interactions characterized by perceived rejection or invalidation (Table 1).

**Table 1.** Characteristics of patients reporting AI-specific psychological stressors in a Nigerian primary care and psychiatry clinic audit, April–August 2025 (N=12).

Characteristic	Patients, n (%)	95% CI^a^
Chatbot interaction distress	7 (58.3)	32.0‐80.7
Job displacement anxiety	3 (25.0)	8.9‐53.2
Health misinformation distress	2 (16.7)	4.7‐44.8
Notable severe outcome		
Chatbot-attributed worsening of suicidal ideation	3 (25.0)	8.9‐53.2

a Wilson Score method. Categories are not mutually exclusive; some patients reported multiple AI-related stressors.

## Discussion

### Principal Findings

AI-related stress affected nearly half of digitally distressed patients, transitioning from theoretical constructs to clinical realities. The finding that three patients reported worsened suicidal ideation following chatbot interactions represents a critical safety signal warranting further investigation.

Pre-existing anxiety and depression may amplify perceived chatbot rejection through negative cognitive biases. In several cases, patients described chatbot responses that they perceived as dismissive or invalidating, which intensified existing feelings of isolation or distress, consistent with the known role of perceived social rejection in suicidality. Digital literacy modulates AI encounter interpretation; lower literacy heightens anthropomorphisation and distress.

Unlike passive social media consumption [1], conversational AI creates bidirectional communication illusions. When systems hallucinate or fail to recognize distress, vulnerable individuals may interpret this as social rejection, a known suicidality risk factor representing a novel form of iatrogenic harm distinct from traditional technostress [8].

Findings align with AIAS dimensions [4] and recent technostress evidence [5,6], and resonate with generative AI safety reports [7,8]. Clinicians should screen for AI interactions in patients with unexplained mood deterioration. Future intake forms should incorporate validated AIAS items.

### Limitations

A small sample (n=28; n=12 AI-stressed) produces wide confidence intervals. Single-center findings may not generalize beyond similar resource-limited settings. Self-reported AI-symptom attribution lacks psychometric rigor, and causal inference is not possible from this retrospective, cross-sectional design. Selection bias is possible. AI stressor categorisation may oversimplify overlapping distress presentations. Despite these limitations, this audit provides preliminary evidence warranting rigorous prospective investigation with validated instruments and larger samples.

### Conclusions

AI stressors are clinically relevant psychiatric presentations. Unregulated AI ’therapist’ tools pose iatrogenic harm demanding urgent regulatory attention. Future research should examine AI stress across larger, cross-national samples using validated instruments to establish prevalence, risk factors, and outcomes essential for evidence-based screening protocols and regulatory frameworks.

## Abbreviations

AI: Artificial Intelligence
AIAS: Artificial Intelligence Anxiety Scale
CI: Confidence Interval
C-SSRS: Columbia–Suicide Severity Rating Scale
NCHRE: National Code of Health Research Ethics
